# Therapy Dog Presence During Chemotherapy in a Day Chemotherapy Unit: A Prospective Pilot Comparative Pre-Post Study

**DOI:** 10.7759/cureus.112198

**Published:** 2026-07-07

**Authors:** Paschalina Tsopa, Michael Kostares, Evangelos Kostares, Gregorios Vestakis, Vasiliki Spyropoulou, Kalliopi Papageorgiou, Athanasia Mpazina, Paul Stampouloglou, Agathi Lekkakou, Maria Piagkou

**Affiliations:** 1 Department of Pulmonology, Metaxa Hospital, Piraeus, GRC; 2 Department of Anatomy, School of Medicine, Faculty of Health Sciences, National and Kapodistrian University of Athens, Athens, GRC; 3 Department of Otolaryngology, Head and Neck Surgery, Metaxa Hospital, Piraeus, GRC; 4 University Department of Oral and Maxilofacial Surgery, Evangelismos General Hospital, Athens, GRC; 5 Department of Psychology/Psychiatry, Metaxa Hospital, Piraeus, GRC

**Keywords:** animal-assisted intervention, day chemotherapy unit, oncology, patient-reported outcomes, therapy dog

## Abstract

Introduction

Therapy dog visits have been introduced in oncology settings as supportive, non-pharmacological interventions. However, evidence regarding their use among adult patients receiving chemotherapy in day hospital settings remains limited. This study evaluated immediate changes in patient-reported symptoms and selected respiratory parameters during chemotherapy in the presence or absence of a therapy dog.

Methods

This prospective pilot comparative pre-post study with room-level allocation was conducted in the Day Chemotherapy Unit of the “Metaxa” Memorial Anticancer Hospital, Greece, between January 19 and March 27, 2026. Seventy adult oncology patients receiving scheduled chemotherapy were included: 35 in the therapy dog group and 35 in the control group. The intervention consisted of the presence of a certified therapy dog and trained handler for approximately 20 to 30 minutes. The control condition consisted of neutral procedural presence without a therapy dog or active supportive intervention. Patient-reported anxiety, relaxation, and dyspnea were assessed before and after the session using 0-10 numerical rating scales. Peripheral oxygen saturation and respiratory rate were also recorded. Change scores were compared between groups, and exploratory adjusted linear regression models were performed.

Results

Compared with the control group, the therapy dog group showed a greater reduction in anxiety and a greater increase in relaxation. Respiratory rate also decreased more in the therapy dog group, although this finding was interpreted as exploratory because respiratory rate differed between groups at baseline. The between-group mean difference in change score was -1.23 for anxiety, 1.83 for relaxation, and -0.49 breaths/min for respiratory rate. No statistically significant between-group differences were observed for dyspnea or peripheral oxygen saturation. Exploratory adjusted analyses were consistent with the unadjusted findings for anxiety, relaxation, and respiratory rate.

Conclusions

Therapy dog presence during chemotherapy was associated with immediate favorable changes in anxiety and relaxation scores in this prospective pilot comparative pre-post study. Findings for respiratory parameters were less consistent and should be interpreted cautiously. These exploratory results support the feasibility of therapy dog presence in a day chemotherapy setting and may inform the design of larger controlled studies.

## Introduction

Cancer treatment is frequently accompanied by psychological distress, anxiety, uncertainty, and treatment-related symptom burden. These difficulties may be particularly relevant during active systemic therapy, when patients are repeatedly exposed to hospital environments, intravenous treatment procedures, waiting time, and concerns regarding disease course and treatment tolerance [1,2]. In this context, supportive oncology increasingly emphasizes the importance of addressing patient-reported symptoms and emotional wellbeing alongside disease-directed treatment [1,3].

Outpatient and day chemotherapy units represent clinically demanding settings in which supportive interventions must be brief, feasible, safe, and compatible with routine care. Patients receiving chemotherapy in these settings may experience anxiety before or during treatment, while also reporting physical symptoms such as dyspnea, fatigue, or general discomfort [2,3]. Because time and staffing resources are often limited, short patient-reported assessment tools may be useful for capturing immediate changes in symptom intensity. Numerical rating scales from 0 to 10 have been widely used in cancer symptom assessment because they are simple, rapid, and suitable for repeated measurements in clinical settings [4].

Animal-assisted interventions, including therapy dog visits, have been introduced in healthcare environments as non-pharmacological supportive interventions. These interventions are generally based on structured or semi-structured interaction with trained animals and handlers, with the aim of improving emotional comfort, reducing distress, and enhancing the patient experience [5,6]. In oncology, animal-assisted interventions have been studied across heterogeneous clinical populations and settings, including adult patients receiving intensive oncological treatment, pediatric oncology patients, palliative care populations, outpatient cancer populations, and mixed cancer samples [7,8]. Existing evidence suggests that such interventions are generally acceptable to patients and may be associated with improvements in mood, anxiety, distress, perceived wellbeing, and treatment experience [5,6].

However, the oncology literature remains methodologically heterogeneous. A systematic review of animal-assisted interventions in oncology found that available studies varied substantially in design, population, intervention format, setting, outcome measurement, and methodological quality [9]. Several studies have reported favorable psychological or experiential outcomes, but many were uncontrolled, included small samples, or assessed mixed populations rather than adult patients receiving chemotherapy in day hospital settings [10,11]. In contrast, findings regarding physiological parameters, such as heart rate, blood pressure, respiratory rate, oxygen saturation, and cortisol, have been less consistent across studies and reviews [12,13].

Evidence specifically addressing adult oncology patients receiving chemotherapy in outpatient or day hospital settings remains limited. One early study evaluated pet therapy among oncological day hospital patients undergoing chemotherapy and reported favorable patient-reported effects, but the broader evidence base in this specific setting remains sparse [14]. Other relevant studies in cancer care, including therapy dog visits among patients undergoing intensive oncological treatment, outpatient patients with brain tumors, women with breast cancer receiving counseling support, and patients with cancer exposed to animal-assisted activities, provide supportive but not definitive evidence because of differences in population, intervention context, outcome assessment, and study design [7,11].

Therefore, additional pilot data are needed to evaluate whether therapy dog presence can be implemented during routine chemotherapy and whether it is associated with immediate changes in patient-reported symptoms and selected respiratory parameters. Such data may help clarify the feasibility and short-term signal of this intervention before larger studies with more rigorous control conditions are undertaken.

The aim of this pilot study was to evaluate the immediate change in patient-reported anxiety during chemotherapy in the presence or absence of a therapy dog in a day chemotherapy unit. Secondary aims were to assess immediate changes in relaxation, dyspnea, peripheral oxygen saturation, and respiratory rate, and to explore whether therapy dog presence was associated with short-term changes in these outcomes under routine clinical practice conditions.

## Materials and methods

Study design and setting

This was a prospective pilot comparative pre-post study with room-level allocation, conducted under routine clinical practice conditions in the Day Chemotherapy Unit of the “Metaxa” Memorial Anticancer Hospital, Greece, between January 19 and March 27, 2026. Data collection began after approval of the study protocol by the Scientific Committee of the hospital. The study was implemented within the framework of the therapy dog program “Parea me Oura” and was designed to evaluate immediate changes in selected patient-reported symptoms and respiratory parameters during chemotherapy in the presence or absence of a therapy dog.

The study was conducted in two predefined chemotherapy rooms within the Day Chemotherapy Unit. The two rooms were of similar bed capacity, layout, view, and general environmental conditions, and both were used as part of the routine operation of the unit. Study procedures were integrated into usual clinical workflow and did not require additional patient visits, modification of chemotherapy administration, or deviation from standard clinical care.

Study population and pre-screening

Patients receiving scheduled chemotherapy in the Day Chemotherapy Unit were assessed for participation during study sessions. Participants were eligible if they were adults, defined as 18 years of age or older, were able to understand and respond to brief numerical rating scales, and provided written informed consent. Exclusion criteria included clinical instability or an acute medical condition requiring urgent intervention, severe cognitive impairment or acute delirium, or reported allergy, fear, discomfort, or any other reason to avoid the presence of a dog.

Before research procedures were initiated, designated clinical staff of the Day Chemotherapy Unit performed a brief safety and tolerability pre-screening as part of routine clinical workflow. This pre-screening was limited to identifying patients who reported allergy, fear, discomfort, or another reason to avoid dog presence. It did not include symptom assessment, psychological evaluation, or discussion of possible benefits of therapy dog presence. Only patients without such contraindications were subsequently approached by the research team for study information and written informed consent.

Allocation of chemotherapy rooms and blinding

The unit of allocation was the chemotherapy room rather than the individual patient. On each study day, two predefined chemotherapy rooms, designated as room A and room B, were used. Patient placement within the chemotherapy rooms followed the usual operational workflow of the Day Chemotherapy Unit and was not modified for research purposes.

After patient information, written informed consent, and baseline assessment had been completed in both rooms, the intervention condition was assigned by simple room-level random allocation. Specifically, either room A or room B was randomly selected to receive the therapy dog visit, while the other room served as the concurrent control room during the same time interval.

Individual patients were therefore not independently randomized. This room-level allocation strategy was selected to preserve routine chemotherapy delivery, avoid interference with clinical care, and minimize disruption of the spatial and operational organization of the unit.

Blinding of patients, healthcare personnel, and research staff was not feasible because of the visible nature of the therapy dog intervention. However, allocation was performed only after completion of baseline assessment in both rooms, and the same structured forms, standardized wording, numerical rating scales, and predefined assessment time points were used in both study conditions.

Intervention

The intervention consisted of the presence of a certified therapy dog accompanied by a trained handler in the chemotherapy room allocated to the intervention condition. The dog and handler remained in the predefined room for approximately 20 to 30 minutes. The visit was low-intensity, non-directive, and non-therapeutic. Participants were not required to interact with the dog, and any interaction occurred only if they wished to engage. No structured psychological, psychoeducational, relaxation, or counseling activity was delivered as part of the intervention.

**Figure 1:**
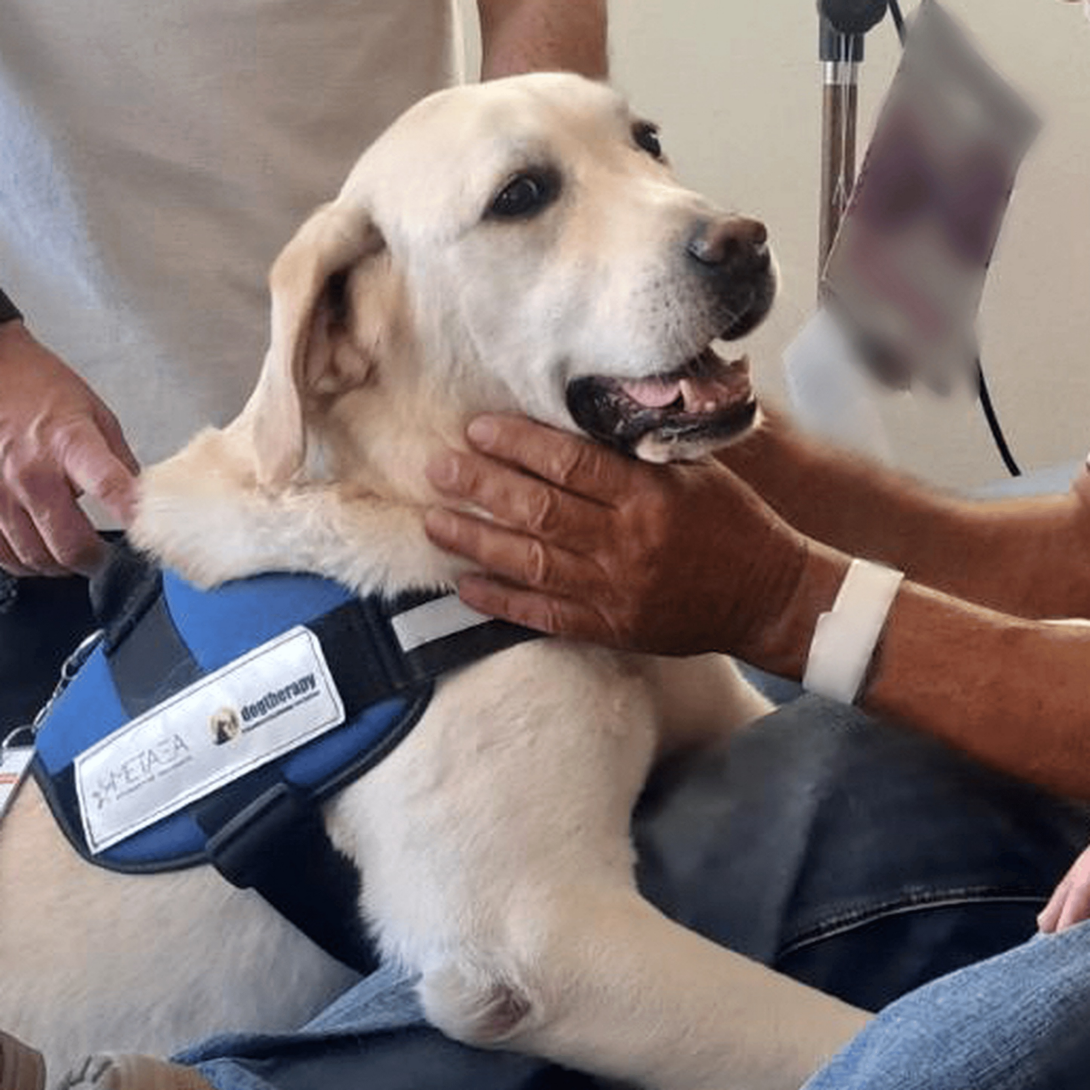
Representative image of therapy dog presence during chemotherapy. The image illustrates the presence of a certified therapy dog in the Day Chemotherapy Unit during routine chemotherapy care.

The therapy dog visit was applied as a supportive environmental presence during chemotherapy and did not replace or modify any component of medical, nursing, or psychological care. Chemotherapy administration and all clinical decisions remained under the responsibility of the treating clinical team. A representative image of therapy dog presence during routine chemotherapy care is shown in Figure 1.

Control condition

The control condition consisted of neutral procedural presence in the chemotherapy room allocated to the control condition for a duration corresponding to the therapy dog visit. Two members of the research team remained present in the control room during the corresponding 20 to 30 minute period. Their role was restricted to neutral procedural presence. They did not provide psychological support, counseling, guided relaxation, emotional exploration, reassurance, personal conversation related to the patients’ condition or treatment, or comments suggesting expected effects of either study condition.

Before study initiation, research team members received standardized instructions regarding their role during the control condition. The purpose of the neutral presence was to standardize the conditions of measurement with respect to time and researcher presence, without introducing an active psychological, supportive, or therapeutic component. The presence of patient companions followed the usual practice of the Day Chemotherapy Unit and was not modified by the study.

Data collection instruments and outcome measures

Data were collected using two study-specific structured assessment forms developed for the purposes of this pilot study: one administered before and one after the session. The forms were designed to be brief, understandable, and feasible for use during active chemotherapy without interfering with routine clinical care. The forms were not intended to constitute a validated multi-item psychometric questionnaire. Instead, they used simple 0-10 numerical rating scales to capture immediate patient-reported symptom intensity. The same wording and response anchors were used before and after the session.

The pre-session assessment form recorded the participant code, date, chemotherapy room, patient-reported symptom scores, self-rated physical condition, presence of a companion, respiratory parameters, supplemental oxygen use, whether chemotherapy infusion had already started, time of assessment, and researcher signature. The post-session assessment form repeated the patient-reported symptom assessment and respiratory measurements and recorded supplemental oxygen use, time of assessment, and researcher signature. Patient-reported outcomes were anxiety, calmness/relaxation, and dyspnea. For anxiety and dyspnea, higher values indicated greater symptom intensity, whereas for calmness/relaxation, higher values indicated greater calmness or relaxation. Overall physical condition was recorded only during the pre-session assessment using a 0-10 scale, where 0 indicated very poor and 10 indicated very good physical condition. Respiratory parameters were recorded by the research team before and after the session and included peripheral oxygen saturation, respiratory rate, and use of supplemental oxygen. Additional descriptive variables included the presence of a companion during the session and whether chemotherapy infusion had already started at the time of the baseline assessment.

The assessment forms were considered appropriate for this pilot study because the items had direct face validity for the intended immediate outcomes, were clinically relevant to the expected short-term effects of the intervention, and could be administered rapidly during chemotherapy. Formal psychometric validation of the full study-specific forms was not performed. Demographic and disease-related variables, including age, sex, primary cancer diagnosis, disease stage, treatment line, and chemotherapy regimen, were not included in the analytic dataset. The primary exploratory outcome was the immediate change in anxiety score between the pre-session and post-session assessments. Secondary exploratory outcomes included the corresponding changes in calmness/relaxation, dyspnea, peripheral oxygen saturation, and respiratory rate.

Data collection procedure

Following safety and tolerability pre-screening, study information, and written informed consent, baseline assessment was performed in both predefined chemotherapy rooms before allocation of the study condition. Room-level random allocation was then performed as described above. The therapy dog and handler entered the room allocated to the intervention condition, while the other room received neutral procedural presence from research team members for the same time interval. The duration of both the intervention and control conditions was approximately 20 to 30 minutes.

Post-session assessment was performed immediately after completion of the intervention or control period, within approximately five to 10 minutes. During questionnaire administration, participants were informed that the questions referred to how they felt at that specific moment and that there were no right or wrong answers. No additional explanations, interpretations, or comments regarding expected effects were provided.

Supervision and process adherence

Session timing and adherence to the planned procedures were monitored by members of the research team. A psychologist supervised the overall procedure to support adherence to the predefined protocol and to monitor that no structured psychological, counseling, or relaxation intervention was delivered as part of either study condition. The psychologist did not administer a therapeutic intervention as part of the study.

A process adherence checklist was used to document whether the study procedures were implemented according to the predefined protocol. The checklist recorded the date, chemotherapy room, study condition, start and end time of the session, whether the session took place in the predefined room, whether the duration was within the planned 20- to 30-minute interval, and whether baseline and post-session assessments were completed at the predefined time points.

For intervention sessions, the checklist documented the presence of the certified therapy dog and trained handler and the absence of guided or structured activity. For control sessions, it documented the presence of research personnel for the corresponding duration, the absence of a therapy dog or comparable intervention, and the absence of organized psychological, psychoeducational, supportive, or other therapeutic activity. Any deviations from the planned procedure were recorded.

Statistical analysis

The final sample size is reported in the Results section. Change scores were calculated as post-session minus pre-session values. For anxiety, dyspnea, and respiratory rate, negative change values indicated a reduction after the session. For calmness/relaxation and peripheral oxygen saturation, positive change values indicated an increase after the session.

Continuous variables were summarized as mean, standard deviation, median, minimum, and maximum values. Categorical variables were summarized as absolute and relative frequencies. Baseline characteristics and baseline outcome values were compared descriptively between the two groups. Welch’s t-test was used for between-group comparisons of continuous variables, and chi-square or Fisher’s exact tests were used for categorical variables, as appropriate.

Within-group pre-post changes were assessed using paired t-tests. Between-group comparisons of change scores were performed using Welch’s two-sample t-test. Because patient-reported outcomes were measured using bounded numerical rating scales and the study had a pilot sample size, Mann-Whitney U tests were also performed as non-parametric sensitivity analyses.

Exploratory adjusted linear regression models were used to examine the association between study group and change scores while accounting for selected baseline factors. In each model, the change score was the dependent variable, and study group was the main independent variable. Models were adjusted for the corresponding baseline value of the outcome, presence of a companion, and whether chemotherapy infusion had already started at baseline. Robust standard errors were used. The study-specific forms were not analyzed as a single psychometric scale, and internal consistency measures such as Cronbach’s alpha were not calculated because the items were not intended to measure one common latent construct. Each item was analyzed separately as an immediate patient-reported or respiratory outcome. Analyses were performed using available paired data for each outcome, and no imputation was performed. Given the pilot nature of the study, findings were interpreted primarily according to the direction and magnitude of change, confidence intervals, and consistency across analyses, rather than as definitive evidence of clinical efficacy.

Ethical and safety considerations

Participation was voluntary, and refusal to participate or withdrawal from the study did not affect routine clinical care. Data were collected in coded form and handled according to applicable data protection requirements. The therapy dog visit followed the safety and hygiene procedures of the therapy dog program and the clinical unit. According to the study protocol, the session would be interrupted in case of patient discomfort or clinical deterioration, and the clinical staff would be informed immediately.

## Results

Study sample and baseline characteristics

The final sample included 70 patients, with 35 patients in the therapy dog group and 35 patients in the control group. Missing data were limited to one baseline anxiety value in the control group and one baseline dyspnea value in the therapy dog group. Accordingly, 69 patients were included in the anxiety change analysis and 69 patients in the dyspnea change analysis.

Baseline characteristics are presented in Table 1. Baseline anxiety and relaxation scores were comparable between groups. Companion presence was more frequent in the therapy dog group, whereas chemotherapy infusion status and supplemental oxygen use were similar between groups. Baseline peripheral oxygen saturation and respiratory rate were higher in the therapy dog group.

**Table 1:** Baseline characteristics of the study sample. Anxiety, relaxation, and dyspnea were assessed using 0-10 numerical rating scales. Peripheral oxygen saturation is reported as percentage. Respiratory rate is reported as breaths per minute. P-values refer to descriptive between-group comparisons at baseline. Welch’s t-test was used for continuous variables. Pearson’s chi-square test was used for categorical variables, except for supplemental oxygen use, for which Fisher’s exact test was used because of small cell counts. Abbreviations: SD, standard deviation; SpO₂, peripheral oxygen saturation.

Characteristic	Control group (n=35)	Therapy dog group (n=35)	p-value
Companion present during the session, n/N (%)	22/35 (62.9)	29/35 (82.9)	0.060
Chemotherapy infusion already started, n/N (%)	26/35 (74.3)	27/35 (77.1)	0.780
Supplemental oxygen use before the session, n/N (%)	2/35 (5.7)	4/35 (11.4)	0.673
Supplemental oxygen use after the session, n/N (%)	2/35 (5.7)	4/35 (11.4)	0.673
Anxiety before the session, mean ± SD	3.85 ± 3.17	4.71 ± 3.19	0.265
Relaxation before the session, mean ± SD	5.77 ± 3.01	5.54 ± 3.36	0.765
Dyspnea before the session, mean ± SD	0.94 ± 2.10	0.26 ± 1.24	0.107
SpO_2 _before the session, mean ± SD	96.23 ± 1.68	97.09 ± 1.29	0.020
Respiratory rate before the session, mean ± SD	13.71 ± 0.93	14.74 ± 1.44	0.001

Pre-session to post-session changes and between-group comparisons

Within-group change scores are presented in Table 2. In the therapy dog group, anxiety decreased and relaxation increased, while respiratory rate also decreased. In the control group, anxiety and respiratory rate remained largely unchanged, while relaxation decreased. Dyspnea and peripheral oxygen saturation showed minimal changes in both groups.

**Table 2:** Within-group change scores by study group. Change scores were calculated as post-session minus pre-session values. Negative values indicate reductions for anxiety, dyspnea, and respiratory rate. Positive values indicate increases for relaxation and peripheral oxygen saturation. Within-group p-values refer to pre-session to post-session comparisons within each study group and were calculated using paired t-tests. Abbreviations: SD, standard deviation; SpO2, peripheral oxygen saturation.

Outcome	Group	Change score, mean ± SD	Within-group p-value
Anxiety	Control	0.12 ± 1.47	0.644
	Therapy dog	-1.11 ± 1.62	<0.001
Relaxation	Control	-0.37 ± 1.66	0.196
	Therapy dog	1.46 ± 2.02	<0.001
Dyspnea	Control	0.09 ± 0.51	0.324
	Therapy dog	-0.09 ± 0.38	0.184
SpO_2_	Control	0.26 ± 0.89	0.095
	Therapy dog	-0.09 ± 1.07	0.638
Respiratory rate	Control	0.06 ± 0.34	0.324
	Therapy dog	-0.43 ± 0.74	0.002

**Figure 2:**
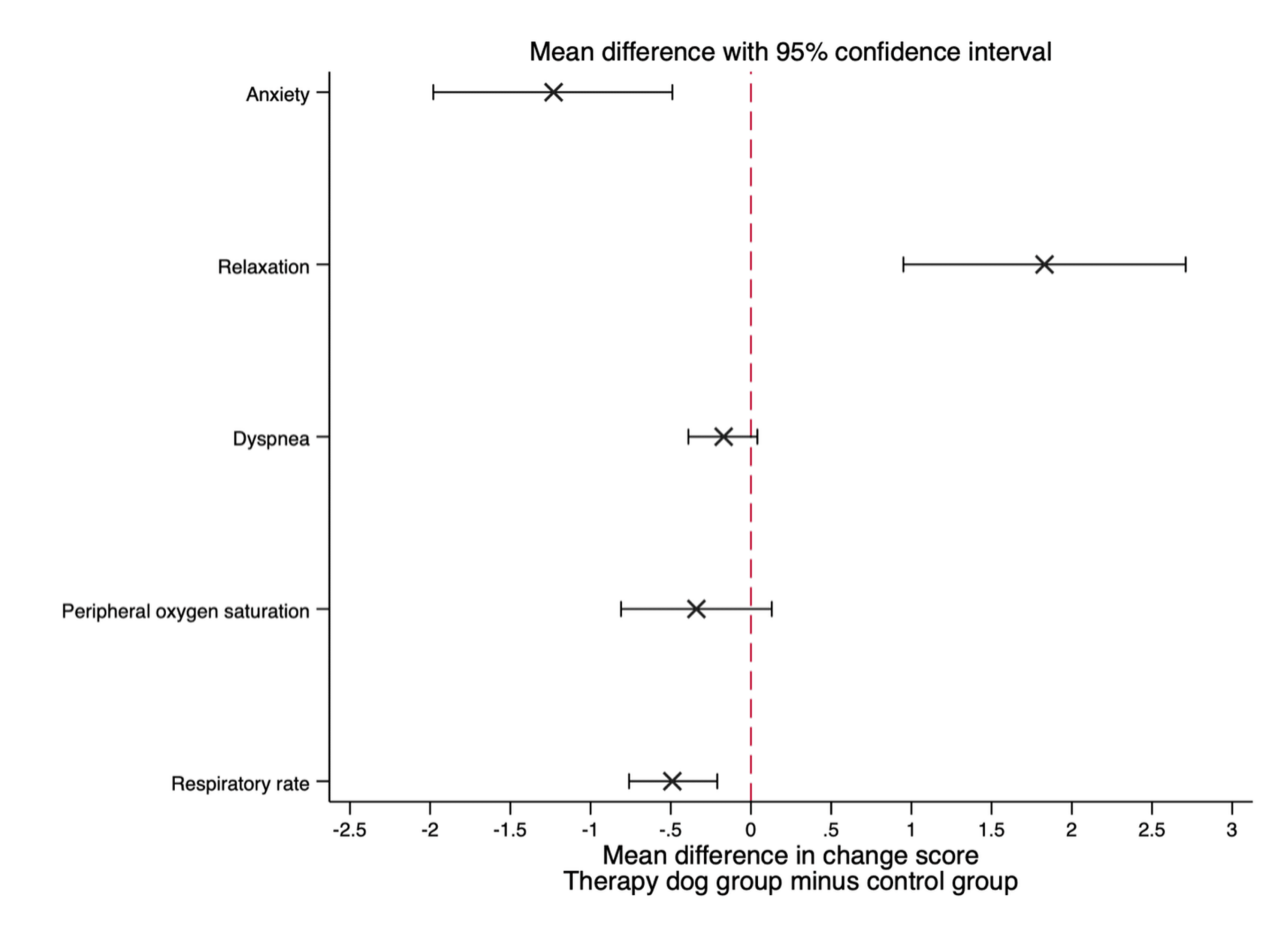
Between-group differences in change scores for patient-reported and respiratory outcomes. Between-group differences are expressed as therapy dog group change minus control group change. Change scores were calculated as post-session minus pre-session values. The therapy dog group showed greater reduction in anxiety and greater increase in relaxation compared with the control group. Respiratory rate also decreased more in the therapy dog group, but this finding should be interpreted cautiously because of baseline imbalance. No statistically significant between-group differences were observed for dyspnea or peripheral oxygen saturation.

Between-group comparisons of change scores are presented in Table 3 and visually summarized in Figure 2. Compared with the control group, the therapy dog group showed a significantly greater reduction in anxiety and a significantly greater increase in relaxation. Respiratory rate also decreased more in the therapy dog group; however, this finding should be interpreted cautiously because respiratory rate differed between groups at baseline. No statistically significant between-group differences were observed for dyspnea or peripheral oxygen saturation.

**Table 3:** Between-group comparison of change scores. Mean difference is expressed as therapy dog group change minus control group change. Change scores were calculated as post-session minus pre-session values. The main between-group comparison was performed using Welch’s two-sample t-test. Mann-Whitney U tests were performed as non-parametric sensitivity analyses. Abbreviations: CI, confidence interval; SpO2, peripheral oxygen saturation.

Outcome	Mean difference	95% CI	Welch’s t-test p-value	Mann-Whitney p-value
Anxiety	-1.23	-1.98, -0.49	0.002	0.001
Relaxation	1.83	0.95, 2.71	<0.001	<0.001
Dyspnea	-0.17	-0.39, 0.04	0.111	0.083
SpO_2_	-0.34	-0.81, 0.13	0.149	0.418
Respiratory rate	-0.49	-0.76, -0.21	0.001	0.001

Exploratory adjusted analyses

**Figure 3:**
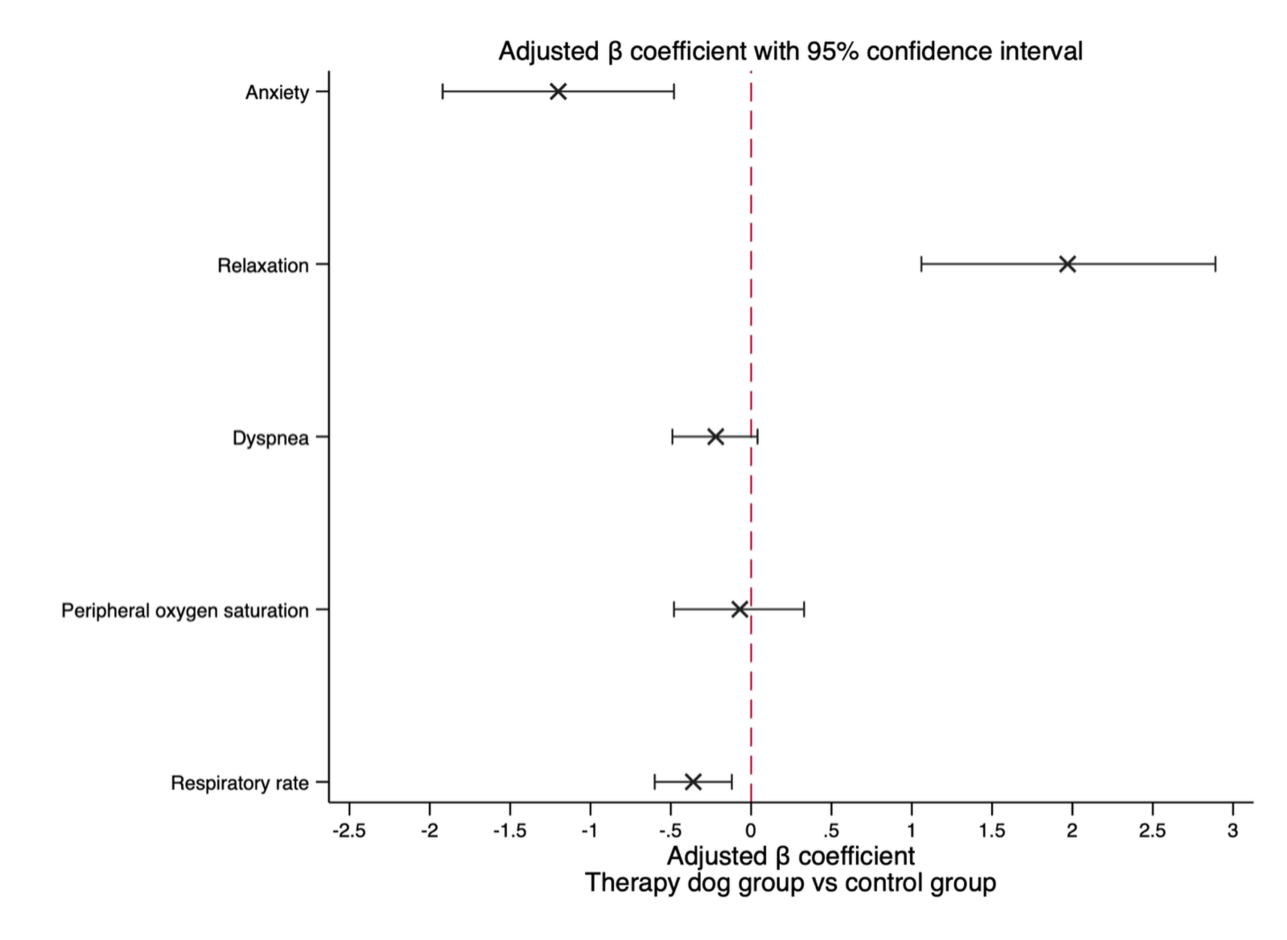
Exploratory adjusted associations between therapy dog presence and change scores. Adjusted β coefficients are shown for the association between therapy dog presence and change scores. The control group was the reference category. Each model was adjusted for the corresponding baseline outcome value, companion presence, and chemotherapy infusion status at baseline, with robust standard errors. Therapy dog presence was associated with greater reduction in anxiety and greater increase in relaxation. A significant adjusted association was also observed for respiratory rate, although this finding was interpreted as exploratory. No statistically significant adjusted association was observed for dyspnea or peripheral oxygen saturation.

Exploratory adjusted linear regression models are presented in Table 4 and visually summarized in Figure 3. After adjustment for the corresponding baseline outcome value, companion presence, and chemotherapy infusion status at baseline, therapy dog presence was significantly associated with greater reduction in anxiety and greater increase in relaxation. A significant adjusted association was also observed for respiratory rate, although this physiological finding was considered exploratory. No statistically significant adjusted association was observed for dyspnea or peripheral oxygen saturation.

**Table 4:** Exploratory adjusted linear regression models for change scores. Adjusted β coefficients represent the adjusted between-group difference in change score for the therapy dog group compared with the control group. Change scores were calculated as post-session minus pre-session values. Each linear regression model included study group as the main independent variable and was adjusted for the corresponding baseline outcome value, companion presence, and chemotherapy infusion status at baseline. Robust standard errors were used. The control group was the reference category. Abbreviations: CI, confidence interval; SpO2, peripheral oxygen saturation.

Outcome	Adjusted β for therapy dog group	95% CI	p-value
Anxiety	-1.20	-1.92, -0.48	0.002
Relaxation	1.97	1.06, 2.89	<0.001
Dyspnea	-0.22	-0.49, 0.04	0.093
SpO_2_	-0.07	-0.48, 0.33	0.718
Respiratory rate	-0.36	-0.60, -0.12	0.004

## Discussion

Principal findings

This pilot comparative pre-post study evaluated the immediate association between therapy dog presence and changes in patient-reported symptoms and selected respiratory parameters during chemotherapy in a day chemotherapy unit. Therapy dog presence was associated with a greater reduction in anxiety and a greater increase in relaxation compared with the control condition. These findings were consistent across unadjusted comparisons, non-parametric sensitivity analyses, and exploratory adjusted regression models.

No statistically significant between-group differences were observed for dyspnea or peripheral oxygen saturation. Respiratory rate decreased more in the therapy dog group; however, this finding should be interpreted cautiously because respiratory rate was higher in the therapy dog group at baseline. Overall, the findings suggest that the immediate signal of therapy dog presence was more evident for psychological outcomes than for respiratory or physiological parameters. Because this was a pilot comparative pre-post study with room-level allocation and without individual randomization or blinding, the findings should be interpreted as exploratory associations rather than causal estimates.

Interpretation of the psychological findings

The observed reduction in anxiety is clinically relevant in the context of active chemotherapy. Patients receiving systemic anticancer treatment may experience distress related to repeated hospital attendance, venous access, treatment uncertainty, fear of adverse effects, and concerns regarding disease course [2]. Supportive oncology therefore emphasizes the assessment and management of distress and patient-reported symptoms alongside disease-directed treatment [3].

The increase in relaxation observed in the therapy dog group is consistent with the proposed supportive role of animal-assisted interventions. The presence of a therapy dog may alter the emotional climate of the treatment environment, provide a pleasant distraction from chemotherapy, promote positive affect, and create a sense of comfort, companionship, and emotional reassurance. Proposed mechanisms of animal-assisted interventions include human-animal bonding, touch, social facilitation, emotional regulation, distraction, and stress modulation [6]. However, the present study was not designed to investigate underlying mechanisms, and these explanations should therefore be regarded as hypothesis-generating.

An important feature of the intervention was its non-directive and non-psychotherapeutic nature. No structured counseling, psychoeducation, guided relaxation, or emotional exploration was delivered. Therefore, the psychological changes observed in the therapy dog group are best interpreted as being associated with therapy dog presence within the chemotherapy environment, rather than with a formal psychological intervention.

Comparison with oncology-specific evidence

The present findings are consistent with the overall direction of the oncology animal-assisted intervention literature, while also addressing a clinical setting in which direct evidence remains limited. A systematic review of animal-assisted interventions in oncology reported substantial heterogeneity in study populations, intervention formats, settings, outcomes, and methodological quality [6]. This variability limits direct comparison between studies but reinforces the need for clinically specific pilot data.

The most directly relevant previous evidence comes from an early study that evaluated a pet therapy intervention among oncological day hospital patients undergoing chemotherapy [14]. That study supports the feasibility and potential benefit of animal-assisted activity in a chemotherapy day hospital setting. The present study adds to this evidence by using a comparative pre-post design, a defined control condition, and immediate pre-session and post-session symptom assessment.

Other oncology studies provide supportive but indirect evidence. One study reported beneficial effects of animal-assisted visits on quality of life among patients receiving combined radiation-chemotherapy regimens [7]. Another small outpatient study involving patients with brain tumors reported preliminary improvements in wellbeing and future-related anxiety after exposure to pet therapy and artist interactions, although the limited sample size and absence of a control group restrict causal interpretation [11]. In addition, a qualitative study among women with breast cancer receiving animal-assisted therapy and counseling support described positive patient experiences related to comfort, engagement, and emotional expression [10].

Additional cancer-specific evidence also supports the relevance of animal-assisted activities in oncology care. A study evaluating animal-assisted activity among patients with cancer found that dog visits were acceptable and valued by participants, although quantitative outcomes did not improve uniformly [15]. Pediatric oncology studies also provide useful implementation data. A pilot study of therapy dog visits among inpatient pediatric and adolescent patients with cancer found that the intervention was feasible and was associated with preliminary reductions in distress-related outcomes [8]. A multisite randomized controlled trial in pediatric oncology suggested benefits particularly for parents and families during the early stages of cancer treatment [16]. More recent pediatric oncology evidence has also supported the feasibility and safety of visiting-dog programs when structured procedures are used [17]. Among children with advanced cancer, animal-assisted interventions have been reported as desirable by both children and parents [18]. Qualitative evidence from pediatric oncology has further suggested that therapy dogs may facilitate communication, comfort, and interaction among patients, families, and nursing staff [19]. Although pediatric data cannot be directly generalized to adult chemotherapy patients, these studies provide relevant information regarding feasibility, acceptability, safety, and implementation in oncology environments.

Psychological versus physiological outcomes

The present study showed clearer findings for anxiety and relaxation than for dyspnea, peripheral oxygen saturation, or respiratory rate. This pattern is consistent with broader animal-assisted intervention research, in which psychological and experiential outcomes appear to be more consistently affected than physiological measures [12,13,20,21].

The absence of a statistically significant between-group difference in peripheral oxygen saturation is clinically plausible. Oxygen saturation is influenced by pulmonary reserve, anemia, cancer burden, cardiopulmonary comorbidity, treatment toxicity, supplemental oxygen use, and baseline oxygenation. In a clinically stable day chemotherapy population, a brief supportive intervention would not necessarily be expected to produce measurable changes in oxygen saturation.

Dyspnea also showed no statistically significant between-group difference. Baseline dyspnea was low, which may have limited the ability to detect improvement. In addition, dyspnea is a multidimensional symptom influenced by physiological, emotional, and disease-related factors. A single brief supportive intervention may reduce anxiety without producing measurable change in perceived breathlessness.

Respiratory rate decreased more in the therapy dog group. This may reflect reduced arousal or increased relaxation, but the finding should remain secondary and cautious. Baseline respiratory rate was higher in the therapy dog group, creating the possibility of regression toward the mean or residual confounding. In addition, baseline peripheral oxygen saturation also differed between groups. Although the adjusted models included the corresponding baseline value of each outcome, the study design does not allow firm conclusions regarding physiological effects.

Previous evidence also supports caution in interpreting physiological outcomes. Some animal-assisted intervention studies have reported favorable changes in parameters such as respiratory rate, blood pressure, heart rate, cortisol, or oxygen saturation, whereas others have found no significant physiological effects [12,13,20,21]. Therefore, the present findings provide stronger support for short-term psychological benefit than for measurable physiological change.

Methodological contribution of the present study

The main contribution of this study is that it examined therapy dog presence during actual chemotherapy delivery in a day chemotherapy unit. Much of the existing literature concerns inpatient settings, palliative care, pediatric populations, counseling contexts, or heterogeneous oncology samples [6,16-19,21]. Direct evidence in adult patients receiving chemotherapy in outpatient or day hospital settings remains limited.

The comparative pre-post design is a strength relative to purely uncontrolled before-after designs. The presence of a control chemotherapy room allowed comparison with patients assessed during the same general clinical workflow but not exposed to the therapy dog. In addition, the control condition was structured as neutral procedural presence rather than psychological support. This does not eliminate bias, but it improves interpretability compared with a single-group pre-post design.

Strengths and limitations

This study has several strengths. First, it was conducted under routine clinical practice conditions, increasing its practical relevance. Second, it included a control condition and used the same assessment structure in both groups. Third, the intervention and control conditions were standardized with respect to session duration, timing of assessment, and wording of measurement instructions. Fourth, the control condition was designed to avoid active psychological or supportive content. Fifth, process adherence was documented using a checklist, including the presence or absence of the therapy dog, session duration, absence of structured activities, and timing of post-session measurements.

Several limitations should be acknowledged. First, this was a pilot study with a relatively small sample size; therefore, the findings should be interpreted as exploratory and hypothesis-generating. Second, allocation occurred at the level of the chemotherapy room rather than at the level of the individual patient. Patient placement followed routine unit workflow and was not modified for research purposes. This approach improved feasibility but may have introduced baseline differences between groups.

Third, blinding was not feasible because of the visible nature of the therapy dog intervention. Patient-reported outcomes may therefore have been influenced by expectation, novelty, preference for animals, or social desirability. This limitation is common in animal-assisted intervention research and should be considered when interpreting subjective outcomes.

Fourth, demographic and disease-related variables, including age, sex, primary cancer diagnosis, disease stage, treatment line, chemotherapy regimen, comorbidities, and baseline clinical status, were not collected in the analytic dataset. This decision was made to keep the assessment brief, minimize patient burden, and avoid disrupting the routine workflow of chemotherapy administration. However, this represents an important methodological limitation. As a result, clinical comparability between groups could not be fully assessed, and potentially relevant clinical confounders could not be included in the adjusted analyses. This is important because anxiety, dyspnea, treatment experience, respiratory rate, and peripheral oxygen saturation may vary according to patient demographics, cancer type, disease burden, comorbidities, and treatment intensity.

Fifth, the study evaluated only immediate pre-session to post-session changes after a single exposure. It does not provide information on durability of effect, repeated exposure, cumulative benefit, patient satisfaction over time, treatment adherence, or longer-term quality of life. Sixth, anxiety, relaxation, and dyspnea were assessed using brief 0-10 numerical rating scales rather than longer validated psychometric instruments. Although these scales were feasible in the clinical setting, they provide limited assessment of complex psychological constructs.

Finally, this was a single-institution study. Generalizability may be limited by local physical layout, staffing patterns, patient population, infection-control procedures, and the structure of the therapy dog program.

Safety and implementation considerations

Safety is central when animal-assisted interventions are implemented in oncology settings. Patients receiving chemotherapy may be immunocompromised because of the disease itself, anticancer treatment, corticosteroids, comorbidities, or treatment-related cytopenias. Potential concerns include allergies, fear or discomfort, animal-related incidents, zoonotic transmission, and contamination of the clinical environment [22-26].

The literature suggests that animal-assisted interventions can be implemented safely when structured protocols are used, although infection-control practices vary across institutions [22,27,28]. In oncology settings, human-animal interaction may offer psychosocial benefits, but these benefits must be weighed against infection-related and safety concerns, especially in patients receiving anticancer treatment [22]. The available literature also shows considerable variation in infection-control practices across hospital animal-assisted intervention programs, supporting the need for standardized procedures, systematic monitoring, and attention to patient, animal, handler, and environmental safety [23]. Expert guidance on animals in healthcare facilities also recommends structured policies addressing animal eligibility, handler responsibilities, hand hygiene, restricted areas, patient selection, and infection-prevention procedures [24,25]. Earlier infection-control literature similarly emphasized the need to define patient suitability, animal suitability, and facility-level infection-control policies before implementation [28]. Survey data from healthcare facilities and infectious disease specialists further indicate that animal-assisted activities are common, but policies vary widely across institutions [26,27].

In the present study, patients were screened for allergy, fear, discomfort, or other reasons to avoid dog presence. The therapy dog visit was optional, non-directive, time-limited, and restricted to a predefined chemotherapy room. The program was implemented according to the safety and hygiene procedures of the therapy dog program and the clinical unit. These safeguards are important for clinical implementation. Hospital-based canine-assisted intervention programs also require consideration of animal welfare, handler training, patient safety, environmental cleaning, staff coordination, and clear procedures for interrupting visits when patient discomfort or clinical deterioration occurs [29].

Future research

Future research should evaluate therapy dog visits in larger, adequately powered studies of adult oncology patients receiving outpatient or day hospital chemotherapy. Randomized or cluster-randomized designs would be useful, particularly if allocation can occur at the level of chemotherapy room, day, or session without disrupting clinical workflow. Attention-matched control conditions should also be considered to distinguish the specific effect of therapy dog presence from nonspecific attention, novelty, or environmental change [16,29,30].

Future studies should collect demographic and clinical variables, including age, sex, cancer diagnosis, disease stage, treatment line, chemotherapy regimen, comorbidities, prior experience with animals, and baseline preference for dogs. These variables would allow assessment of group comparability, adjustment for potential confounders, and subgroup analyses.

Outcome assessment should combine brief symptom scales with validated instruments for state anxiety, distress, mood, quality of life, and patient experience. Physiological outcomes may be included, but they should be treated as secondary or exploratory unless the study is specifically powered for those endpoints. Repeated assessments across multiple chemotherapy sessions would help determine whether the observed psychological changes are transient, reproducible, or cumulative.

Qualitative research may also be valuable. Patient interviews could clarify how therapy dog presence modifies the experience of chemotherapy, which patients perceive the greatest benefit, and which aspects of the interaction are most meaningful. Staff perspectives would also be useful for evaluating workflow, safety, feasibility, sustainability, and acceptability among healthcare professionals [19,29,30].

## Conclusions

In this pilot comparative pre-post study, therapy dog presence during chemotherapy was associated with immediate reduction in anxiety and increased relaxation compared with a control condition. No statistically significant between-group differences were observed for dyspnea or peripheral oxygen saturation. Respiratory rate decreased more in the therapy dog group, but this finding should be interpreted cautiously because of baseline differences. The findings are consistent with the broader animal-assisted intervention literature, which suggests more consistent effects on psychological and experiential outcomes than on physiological parameters. Larger controlled studies are needed to determine whether therapy dog visits produce reproducible and clinically meaningful benefits for adult oncology patients receiving chemotherapy in day hospital settings.
